# The precursor for nerve growth factor (proNGF) is not a serum or biopsy-rinse biomarker for thyroid cancer diagnosis

**DOI:** 10.1186/s12902-019-0457-1

**Published:** 2019-11-27

**Authors:** Christopher W. Rowe, Sam Faulkner, Jonathan W. Paul, Jorge M. Tolosa, Craig Gedye, Cino Bendinelli, Katie Wynne, Shaun McGrath, John Attia, Roger Smith, Hubert Hondermarck

**Affiliations:** 1 School of Medicine and Public Health, University of Newcastle, Newcastle, Australia; 2 Department of Endocrinology, John Hunter Hospital, Locked Bag 1 HMRC, Newcastle, 2310 Australia; 3 Hunter Medical Research Institute, New Lambton Heights, Australia; 4 School of Biomedical Sciences and Pharmacy, University of Newcastle, Newcastle, Australia; 5 Department of Surgery, John Hunter Hospital, Newcastle, Australia; 6 Department of Medical Oncology, Calvary Mater Newcastle, Waratah, Australia; 7 Clinical Research Design, IT, and Statistical Support Unit, Hunter Medical Research Institute, Newcastle, Australia

**Keywords:** Thyroid Cancer, proNGF, Biomarker, Serum, Biopsy-rinse

## Abstract

**Background:**

Nerves and neurotrophic growth factors are emerging promoters of cancer growth. The precursor for Nerve Growth Factor (proNGF) is overexpressed in thyroid cancer, but its potential role as a clinical biomarker has not been reported. Here we have examined the value of proNGF as a serum and biopsy-rinse biomarker for thyroid cancer diagnosis.

**Methods:**

Patients presenting for thyroid surgery or biopsy were enrolled in separate cohorts examining serum (*n* = 204, including 46 cases of thyroid cancer) and biopsy-rinse specimens (*n* = 188, including 26 cases of thyroid cancer). ProNGF levels in clinical samples were analysed by ELISA. Univariate and multivariate statistical analyses were used to compare proNGF levels with malignancy status and clinicopathological parameters.

**Results:**

ProNGF was not detected in the majority of serum samples (176/204, 86%) and the detection of proNGF was not associated with thyroid cancer diagnosis. In the few cases where proNGF was detected in the serum, thyroidectomy did not affect proNGF concentration, demonstrating that the thyroid was not the source of serum proNGF. Intriguingly, an association between hyperthyroidism and serum proNGF was observed (OR 3.3, 95% CI 1.6–8.7 *p* = 0.02). In biopsy-rinse, proNGF was detected in 73/188 (39%) cases, with no association between proNGF and thyroid cancer. However, a significant positive association between follicular lesions and biopsy-rinse proNGF was found (OR 3.3, 95% CI 1.2–8.7, *p* = 0.02).

**Conclusions:**

ProNGF levels in serum and biopsy-rinse are not increased in thyroid cancer and therefore proNGF is not a clinical biomarker for this condition.

## Background

Thyroid cancer is a common endocrine malignancy. In the United States, an average of 14 new cases per 100,000 person are diagnosed each year, with an annual increase in incidence of 3.6% since 1974 [1]. This rise is due to a combination of increased diagnosis of clinically indolent cancers, and a true but small increase in aggressive cases, with a corresponding small rise in incidence-based mortality [1]. Thus, the timely diagnosis of clinically significant thyroid cancers is an important public health priority. These cancers must be distinguished from more common benign thyroid nodules, detected by ultrasound in 19–35% of adults [2]. At present, the diagnostic evaluation of nodular thyroid disease includes thyroid ultrasound and fine-needle aspiration (FNA) biopsy [3]. Importantly, no current technique can accurately predict clinically significant cancers from indolent thyroid cancers, as demonstrated by the “epidemic” of non-lethal papillary thyroid cancer in countries that have introduced neck ultrasound screening programs [4]. Therefore, blood-based and biopsy-based biomarkers are needed to refine the diagnosis and prognosis of thyroid cancers [5–7].

Nerves and neurotrophic growth factors are emerging promoters of tumorigenesis and are increasingly regarded as potential biomarkers and therapeutic targets in oncology [8, 9]. The precursor for nerve growth factor (proNGF) has recently been shown to be overexpressed in thyroid cancer compared to benign thyroid tissues, suggesting utility as a discriminator in diagnostic testing [10]. ProNGF is a soluble 246 amino acid pro-peptide, transcribed from the nerve growth factor (*NGF)* gene on chromosome 1p13. ProNGF is cleaved into nerve growth factor (NGF) by tissue proconvertases such as furin and matrix metalloproteinases [11]. ProNGF has an established role in neural development in the foetus [12], and acts on neurons through interaction with specific NGF receptors to promote neural survival and differentiation, or apoptosis [11]. Interestingly, proNGF and its receptors have been associated with progression and aggressiveness of several cancers, including breast [13, 14], prostate [15], and melanoma [16]. In thyroid cancer, in addition to proNGF overexpression [10], the upregulation of proNGF/NGF receptors (the tyrosine kinase TrkA, the neurotrophin receptor p75^NTR^ and the pro-neurotrophin receptor sortilin) has been reported [17], suggesting a role for proNGF in thyroid carcinogenesis and a potential value as a diagnostic or prognostic biomarker.

In the present study, we hypothesized that the overexpression of proNGF may lead to an increased level of proNGF in the serum of patients with thyroid cancer, as compared with benign thyroid conditions, and might represent a useful biomarker for diagnosis and risk stratification of nodular thyroid disease. Further, we hypothesized that proNGF protein may also be detected in the needle-rinse of thyroid biopsy specimens, in a similar manner to the needle-rinse techniques used for assaying for thyroglobulin [18] and calcitonin [19]. Here we report the results of studies evaluating these hypotheses in nodular thyroid disease.

## Methods

### Patients and samples

This study was approved by the Hunter New England Local Health District Human Research Ethics Committee (HREC/16/HNE/247), and all participants provided written informed consent. To collect serum, we conducted a prospective nested cohort study, enrolling patients undergoing thyroid surgery or thyroid fine-needle aspiration biopsy for investigation or management of thyroid disease. To collect biopsy material, we conducted a prospective cohort study of patients referred for thyroid FNA biopsy at a single high-volume clinic.

In both cohorts, patients were followed after bio specimen collection to obtain a final diagnosis of their thyroid disease based on histopathology (surgical patients) or a composite clinical assessment (clinical, ultrasound and FNA biopsy) for non-surgical patients. Relevant clinical data were extracted from the medical record to correlate levels of proNGF with age, sex, presence of hyperthyroidism (defined as thyroid stimulating hormone (TSH) level < 0.1 mIU/L), and thyroid histopathology.

### Serum study

Prior to thyroid surgery or thyroid biopsy, serum was drawn into a serum separator tube (surgery-only patients) or plain serum tube (biopsy-first patients), centrifuged to separate, then aliquoted and frozen at − 80 °C. Serum samples were assayed using a proNGF enzyme-linked immunosorbent assay (ELISA) (see below) on the first or second freeze-thaw cycle only. Samples were run in triplicate at 1:20 dilution (to minimize matrix effects, as recommended by the manufacturer), with positive results confirmed on a second plate; and run with an in-house quality control (QC) samples of serum spiked with recombinant human proNGF (Biosensis Pty Ltd., Adelaide, Australia). 4-parameter logistic regression curves were fit using GraphPad Prism (v7.0 California, USA). All results above the limit of detection of 0.05 ng/mL (a functional limit of 1 ng/mL allowing for 20x dilution) were reported as proNGF positive.

### Biopsy rinse study

Consecutive consenting adults over 18 years with a thyroid nodule graded as ‘Low-’, ‘Intermediate-’ or ‘High-risk’, according to the Sonographic Pattern stratification of the 2015 American Thyroid Association [3], were prospectively enrolled. Each nodule was biopsied using a 25 g needle with capillary action technique. After expulsion of the cellular material for diagnostic cytopathology, the needle was rinsed with 0.5 mL phosphate-buffered saline at 4 °C with the addition of protease inhibitors (cOmplete Mini, Roche, Manneheim Germany, Catalogue number 046931590011, 1 tablet per 10 mL), with subsequent refrigerated centrifugation to pellet red blood cells and insoluble debris. The supernatant containing solubilised proteins was removed and stored at − 80 °C prior to ELISA, performed without dilution in duplicate (due to constraints on sample volume) and analysed as above. This ‘needle-rinse’ technique is established as a sensitive method of detecting the thyroid-specific proteins thyroglobulin (an established biopsy-based tumour marker for metastatic thyroid cancer) [18] and calcitonin (an established biopsy-based tumour marker for medullary thyroid cancer) [19], and has the advantage of preserving cytological material for diagnostic purposes whilst potentially yielding additional information from the solubilised proteins. All results above the limit of detection of 0.05 ng/mL were reported as proNGF positive.

### ProNGF ELISA validation

ProNGF was quantified using a human enzyme-linked immunosorbent assay kit (BEK-2226; Biosensis Pty Ltd., Adelaide, Australia), with wells coated with an antibody raised against the N-terminal precursor domain of human proNGF. Heterophilic antibody blockers were added as recommended by the manufacturer [20] to a final concentration of 38 μg/mL.

Performance of the proNGF ELISA was confirmed using spike and recovery and linearity of dilution experiments (Additional file 1: Table S1). A mean of 96% spike recovery was obtained (range 80–128%) when assayed in the presence of supplied heterophilic antibody blockers. Mean recovery of the in-house QC sample, which was assayed across all plates, was 98 ± 22% for serum, and 117 ± 20% for rinse. The between assay coefficient of variation was 20%, and the within-assay coefficient of variation (between wells) was 3.6 ± 2.9%.

For serum, no difference in rates of proNGF detection were observed in samples collected in serum-separator (16% positive, *n* = 95) vs plain serum tubes (12% positive, *n* = 109) (unadjusted *p* = 0.42; adjusted for age, sex and thyroid hormone status *p* = 0.79), suggesting that proNGF is not sequestered in the gel layer of a serum separator tube. Additionally, no difference in levels of proNGF detection were observed in samples stored for more than 12 months (13%, *n* = 117), compared to less than 12 months (21%, *n* = 87) (unadjusted *p* = 0.21, adjusted for age, sex, thyroid cancer and hyperthyroidism *p* = 0.79), suggesting that endogenous proNGF is stable at − 80 °C for at least 12 months.

### Statistical analysis

Power calculations were based on pilot data, using a power of 0.8 and two sided alpha of 0.05. For the serum study, to detect a 3-fold increase in proNGF levels in patients with cancer, above the background detection of proNGF cleavage products in 6–10% of healthy sera [21], 46 cases and 160 controls were required. For the biopsy study, the diagnostic performance of proNGF in histological specimens generated an area under the ROC curve of 0.94 [10]. Conservatively assuming that our tests generate an AUC ROC of 0.85, and that the minimum clinically significant value is 0.7, 28 cases with thyroid cancer and 124 benign nodules were required.

Between group comparisons were assessed categorically using the Pearson’s Chi-square test, and continuously using the Wilcoxon Rank-Sum test, with multiple logistic regression to assess for potential interaction from clinical variables. Analyses were performed using the statistical software package Stata (version 14, Statacorp, Texas, USA).

Between 2014 and 2017, 204 patients with thyroid diseases were enrolled in the serum cohort (46 cases of thyroid cancer and 158 cases of benign thyroid conditions); and between 2016 and 2018, 183 patients with 188 nodules were enrolled in the biopsy cohort (26 cases of thyroid cancer and 162 benign nodules). Demographic and clinical information regarding the two cohorts are presented in Table 1.

**Table 1:** Patient Demographics

	Serum study		Biopsy study	
*N*	204		188	
Female (n, %)	162 (79%)		153 (81%)	
Age, years (mean ± SD)	53 ± 16		55 ± 15	
TSH, mIU/L (mean ± SD)	1.1 ± 1.3		1.3 ± 0.88*	
TSH < 0.1 mIU/L (*n*, %)	28 (14%)		1 (1%)	
Nodule Diagnosis				
Thyroid cancer (*n*, %)	46 (24%)		26 (14%)	
Papillary	36		19	
Follicular/Hurthle carcinoma	8		5	
Anaplastic	1		2	
Medullary	1		0	
Benign nodule (*n*, %)	158 (76%)		162 (86%)	
Nodular goitre	109		139	
Follicular/Hurthle adenoma	13		15	
Graves’	25		1	
Lymphocytic	9		7	
Normal	2		0	
Diagnostic basis	Histology	Follow up	Histology	Follow up
Thyroid Cancer	46	0	25	1#
Benign nodule	82	74	41	121

*TSH data not available for 9 cases. #One patient with anaplastic cancer did not undergo thyroidectomy

## Results

### Serum proNGF concentration is not associated with thyroid cancer

Overall, 176/204 (86%) of serum samples were negative for proNGF. In the remaining 14% of serum samples in which proNGF could be detected, median serum proNGF concentration was 6.2 ng/mL (IQR 4.2–12.4 ng/mL). With respect to the primary hypothesis, positive serum proNGF was detected in 6/46 (13%) cases of thyroid cancer, and in 22/158 (14%) benign samples (*p* = 0.97). The 6 positive results in the malignant group occurred in 5/36 papillary thyroid cancers and 1/8 follicular/hurthle-cell thyroid cancers (Additional file 2: Table S2). Median proNGF levels were not significantly different between benign and malignant cohorts (Table 2). Therefore proNGF is not a serum biomarker for thyroid cancer diagnosis.

**Table 2:** Serum proNGF levels, grouped by demographic and disease classification. Differences between groups are assessed using Pearson’s Chi-Square test (binary classification at the 1 ng/mL limit of detection) and Wilcoxon Rank-Sum test (continuous)

Category	Serum proNGF (dichotomised)		Serum proNGF (continuous)		Multiple logistic regression*	
	*n* > 1 ng/mL/n in group	*p*-value	Median (IQR)	*p*-value	OR (95% CI)	*p*-value
Overall	28/204 (14%)					
*By malignancy status*		0.88		0.97	1.0 (0.4–2.7)	0.98
-Thyroid cancer	6/46 (13%)		0 (0–0)			
-Benign thyroid diseases	22/158 (14%)		0 (0–0)			
*By thyroid hormone status*		0.002		0.002	3.3 (1.3–8.7)	0.02
-Hyperthyroid	9/28 (32%)		0 (0–1.74)			
-Euthyroid	19/176 (11%)		0 (0–0)			
*By follicular lesion*		0.93				
-Present	3/21 (14%)		0 (0–0)	0.80	1.1 (0.3–4.2)	0.88
-Absent	25/183 (14%)		0 (0–0)			
*By age*		0.20		0.11	1.0 (1.0–1.0)	0.18
-Age < 55	18/108 (17%)		0 (0–0)			
-Age ≥ 55	10/96 (10%)		0 (0–0)			
*By sex*		0.53		0.54	0.7 (0.3–1.8)	0.44
-Female	21/162 (13%)		0 (0–0)			
-Male	7/42 (17%)		0 (0–0)			

Categorical variables evaluated with Pearson’s Chi-square, and continuous variables with the Wilcoxon RankSum test. *Binary outcome variable is proNGF > 1 ng/mL, adjusting for age (continuous) presence of cancer, presence of hyperthyroidism, presence of follicular lesion, and female sex

**Fig. 1:**
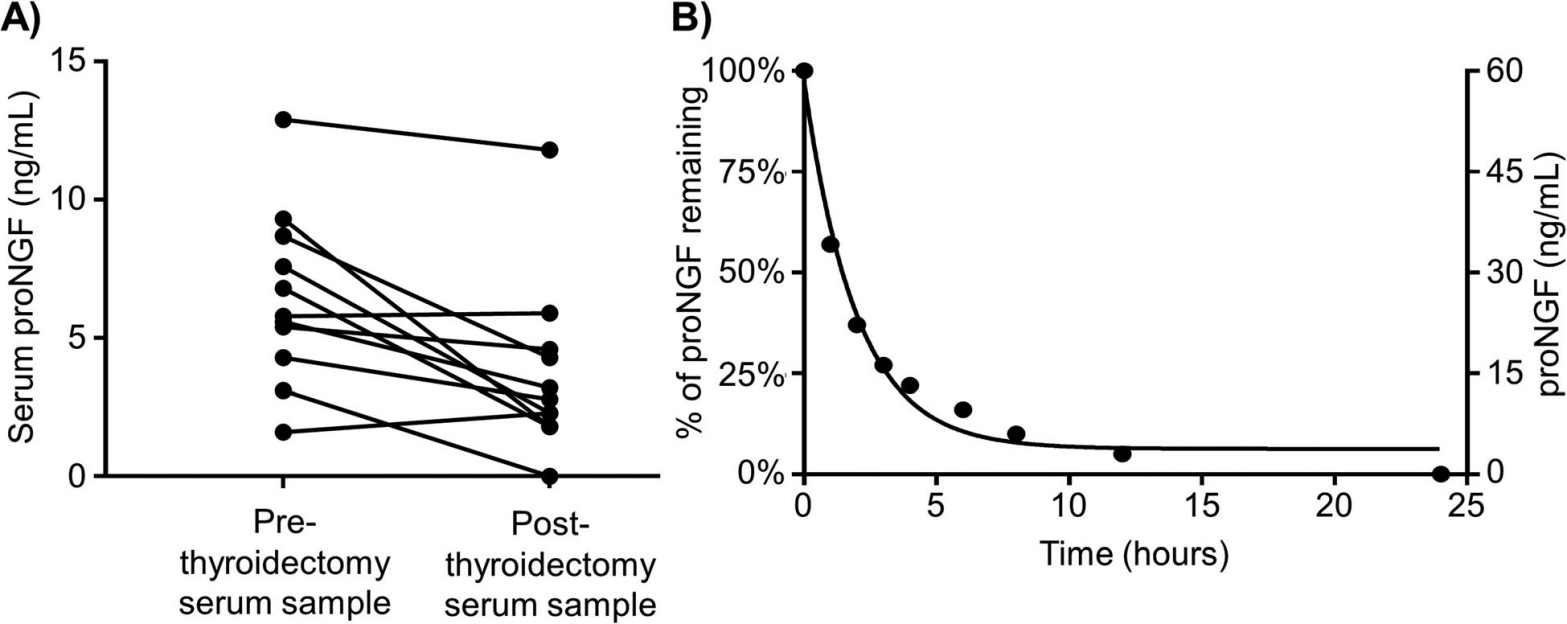
ProNGF serum levels after thyroidectomy and half-life. **a** Change in serum proNGF following total thyroidectomy**.** Pre- and post- thyroidectomy serum samples were available for 11 cases where pre-operative serum proNGF was detectable. No significant difference was detected between pre- and post- thyroidectomy levels of proNGF. **b** ProNGF in vitro half-life. Aliquots of serum negative for proNGF was spiked with 20 ng/mL recombinant proNGF dissolved in Assay Diluent A (Biosensis, Australia) in a 1:1 ratio, then incubated at 37 °C for increments of 24 h, then assayed at 1:20 dilution with Heterophilic Blocking Antibody (BL-003-1000). An exponential decay curve was fitted, giving an estimated in-vitro half-life in serum of 1.5 h. Similar results were obtained using phosphate-buffered-saline as diluent

Post-thyroidectomy sera (range 2–14 days post-operative) were available for analysis, with 11 cases positive for proNGF, and 20 cases negative for proNGF. Figure 1a shows 10/11 (91%) cases with detectable pre-operative proNGF (median 5.8 ng/mL, IQR 4.9–8.2) remained positive in the post-thyroidectomy sample (median 2.8 ng/mL, IQR 2.1–4.5). 18/20 (90%) cases with negative pre-operative proNGF had a concordant post-thyroidectomy sample. As the in-vitro serum half-life of proNGF was determined to be 90 min (Fig. 1b), our results suggest that the proNGF detected in the serum was not of thyroid origin.

### Serum proNGF may be associated with hyperthyroidism

**Fig. 2:**
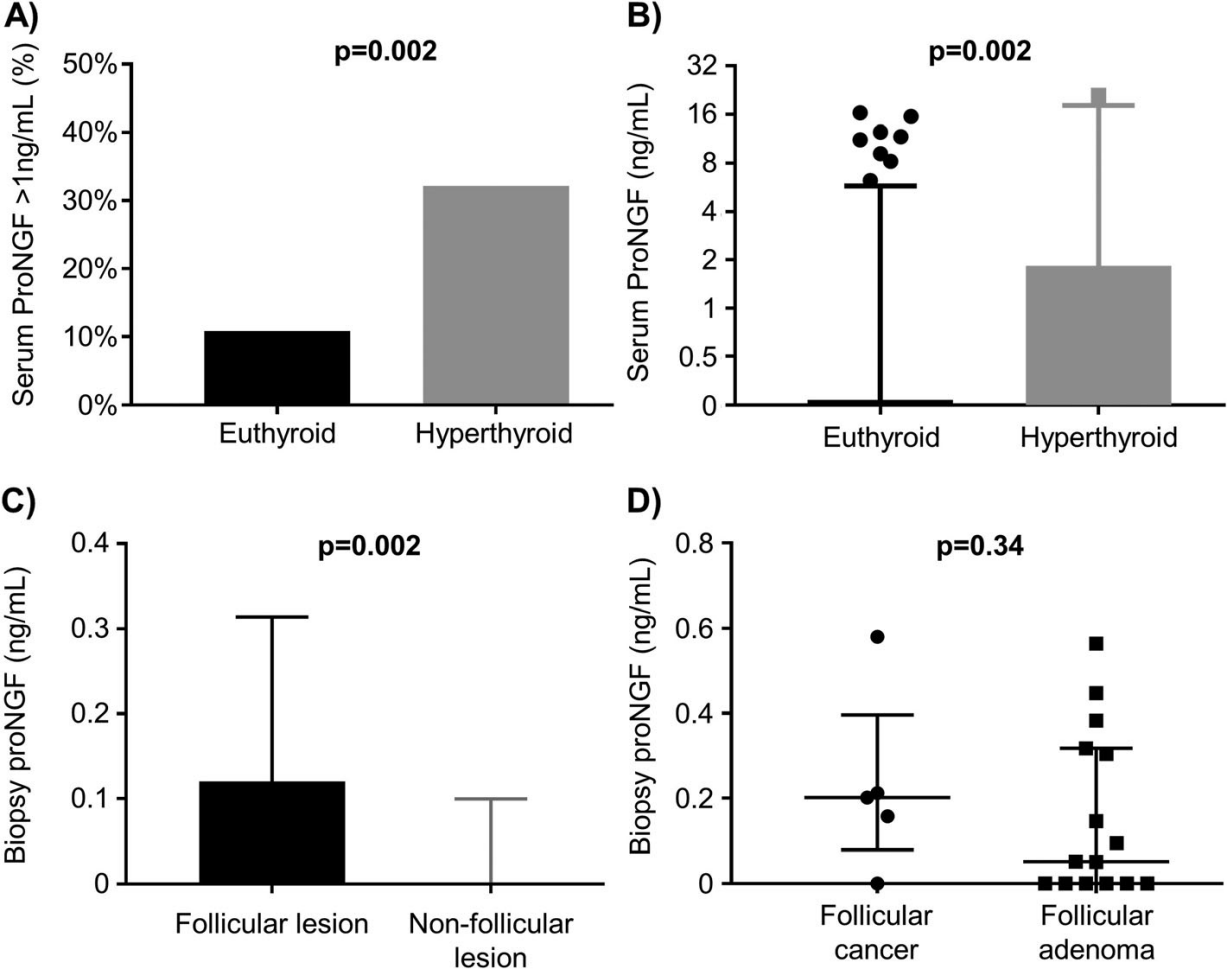
Subgroup analysis of proNGF. **a** Bar graph showing detection of serum proNGF as a binary variable, stratified by hyperthyroid status. See Table 2 for details. **b** Box (interquartile range) and whisker (5–95% range) demonstrating detection of serum proNGF as a continuous variable, stratified by hyperthyroid status. See Table 2 for details. **c** Box (interquartile range) and whisker (5–95% range) graph showing concentration of proNGF in biopsy rinse, stratified the presence of follicular lesions. See Table 3 for details. **d** Scatter plot (with median and interquartile range overlaid) showing concentration of proNGF in biopsy-rinse, stratified by malignant status of follicular lesions. See Table 3 for details

Analysis of serum proNGF data was undertaken in subgroups of hyperthyroidism, age and sex (Table 2). ProNGF was above 1 ng/mL in 9/28 (32%) of hyperthyroid cases, compared to 19/176 (11%) of euthyroid cases (Pearson’s chi-square *p* = 0.002, Fig. 2a). ProNGF levels were higher in sera from patients who were hyperthyroid at the time of sampling (TSH < 0.1mIU/L) than in those who were euthyroid (proNGF interquartile range 0–1.76 ng/mL vs 0-0 ng/mL respectively, Wilcoxon Rank-Sum p = 0.002, Fig. 2b). No difference was observed in proNGF levels based on age or sex or follicular lesions. Multiple logistic regression was performed to assess the interaction of thyroid cancer, age, sex, follicular lesion and hyperthyroid status on serum proNGF levels (Table 2). The odds ratio for serum proNGF > 1 ng/mL (compared to ≤1 ng/mL, the assay limit of detection) in the presence of hyperthyroidism, holding other variables constant, was 3.3 (95% CI 1.6–8.7 *p* = 0.02). No association was found with the other parameters, and there was no confounding observed.

### Biopsy rinse proNGF is not associated with thyroid cancer

Overall, 73/188 (39%) of biopsy-rinse specimens were positive for proNGF. Median proNGF concentration in positive samples was 0.15 ng/mL (IQR 0.1–0.2 ng/mL). With respect to the primary hypothesis, biopsy-rinse proNGF was detected in 12/26 (46%) nodules with thyroid cancer, and in 61/162 (38%) benign nodules (*p* = 0.41). The 12 positive results in the malignant group occurred in 7/19 papillary thyroid cancers (37%) and 4/5 follicular/Hurthle-cell thyroid cancers (80%). Median proNGF levels were not significantly different between benign and malignant thyroid nodules (Table 3). Individual patient characteristics for the 73 cases of detectable proNGF are presented in Additional file 3: Table S3.

**Table 3:** Biopsy rinse proNGF levels, stratified by nodule diagnosis

Category	Biopsy-rinse proNGF (dichotomised)		Biopsy-rinse proNGF (continuous)		Multiple logistic regression*	
	*n* > 0.05 ng/mL/n in group	*p*-value	ng/mL Median (IQR)	*p*-value	OR (95% CI)	*p*-value
Overall	73/188 (39%)		0 (0–0.12)			
*By malignancy status*		0.41		0.11	1.3 (0.5–3.1)	0.57
-Thyroid cancer	12/26 (46%)		0 (0–0.20)			
-Benign nodule	61/162 (38%)		0 (0–0.10)			
*By follicular lesion*		0.01		0.002	3.3 (1.2–8.7)	0.02
-Present	13/20 (65%)		0.12 (0–0.31)			
-Absent	60/168 (36%)		0 (0–0.11)			
*By age*		0.61		0.82	1.0 (1.0–1.0)	0.87
-Age < 55	39/96 (41%)		0 (0–0.11)			
-Age ≥ 55	34/92 (37%)		0 (0–0.13)			
*By sex*		0.35		0.33	0.7 (0.2–2.3)	0.35
-Female	57/153 (37%)		0 (0–0.11)			
-Male	16/35 (46%)		0 (0–0.17)			

Categorical variables evaluated with Pearson’s Chi-square, and continuous variables with the Wilcoxon RankSum test. *Binary outcome variable is proNGF > 0.05 ng/mL, adjusting for age (continuous) presence of cancer, presence of follicular lesion and female sex

### Biopsy rinse proNGF may be associated with follicular lesions

Analysis of the biopsy-rinse proNGF cohort was undertaken in subgroups of age, sex and follicular lesions (including follicular adenoma, Hurthle cell adenoma, follicular carcinoma and Hurthle cell carcinoma) (Table 3). Insufficient patients were hyperthyroid at the time of biopsy (as hyperfunctioning nodules have a low risk of malignancy and are rarely biopsied) for analysis of this cohort by hyperthyroid status (see Table 1). ProNGF levels were higher from follicular lesions (median 0.12 vs 0 ng/mL, *p* = 0.002) compared to other nodules (Fig. 2c). However, proNGF was detected at similar rates in both benign (9/15, 60%) and malignant (4/5, 80%) follicular lesions, suggesting that this is not a useful discriminative marker for follicular thyroid cancer (*p* = 0.42), and there was no difference in concentration of proNGF between benign and malignant follicular lesions (Fig. 2d, *p* = 0.34). Multiple logistic regression, dichotomizing biopsy proNGF at 0.05 ng/mL (negative/positive) as the dependent variable, and including model variables of age, sex, follicular lesion and malignant status, continued to demonstrate an association between proNGF and follicular lesions (odds ratio 3.3, 95% CI 1.2–8.7, *p* = 0.02), holding other parameters constant (Table 3). No other parameter showed significant association and there was no evidence of confounding.

## Discussion

The over-diagnosis of clinically indolent thyroid cancer necessitates the development of biomarkers that better predict future disease aggressiveness to allow clinicians and patients to match treatment intensity with disease risk. An inexpensive protein-based biomarker in serum, or as an adjunct to needle biopsy, represents an attractive translational biomarker, and pilot data for proNGF suggested a possible utility for this protein in this role [10].

This present study, reporting proNGF evaluation in a large cohort of serum and biopsy material, found no difference in levels of proNGF between cases of thyroid cancer and other thyroid diseases. The study was adequately powered to detect clinically meaningful differences in proNGF levels. A smaller difference is unlikely to be clinically useful as a biomarker of thyroid malignancy. Therefore, the increased level of proNGF tissue expression in thyroid cancer previously observed [10] does not result in an increased proNGF concentration in the serum of thyroid cancer patients, or provide a sufficiently discriminatory level of proNGF in biopsy specimens, demonstrating that, based upon these data, serum and biopsy-rinse proNGF is not of clinical value for the diagnosis or prognosis of thyroid cancer.

A prior study has examined the presence of proNGF in sera of 20 patients with and without diabetic retinopathy, using Western blotting [22]. They found that a small subset of patients with diabetic retinopathy had detectable serum proNGF, although exact quantification was not possible due to the limitations of Western blot methodology. A study of 227 patients with autoimmune diseases measured LIP1 and LIP2 (short cleavage products of proNGF peptide) in serum using ELISA, with rates of positivity in control serum of 6 and 10% respectively. These findings are concordant with the present study [21]. Contrastingly, a recent study of 116 patients (77 with Parkinson’s Disease and 39 healthy controls) detected serum proNGF using ELISA in all participants at a very low level, in the range of 0.085–0.122 ng/mL, 10–100 fold lower than detected in our study [23]. Together, these data and our study indicate generally low levels of proNGF in human sera across a variety of conditions. There were no prior data on the levels of proNGF in thyroid biopsy specimens.

The majority of patients with thyroid cancers included in both the serum and needle-rinse studies were diagnosed with the papillary subtype, and it is possible that other subtypes (follicular, medullary) may have different systemic expression patterns of proNGF. However, previous immunohistochemistry data demonstrated that the strongest overexpression of proNGF was in papillary cancers [10], so any positive signal would have been expected in this group. Medullary thyroid cancers, derived from neuro-endocrine parafollicular C-cells, may be more likely to secrete a neurotrophins such as proNGF [24, 25]. However, medullary tumors have established and highly sensitive serum biomarkers: calcitonin and carcino-embryonic antigen [26], and therefore the clinical utility of additional markers may have limited translational value.

Intriguingly, our study observed an association between serum proNGF and hyperthyroidism, which has not previously been described in humans. However, studies in mice have shown that administration of the thyroid hormones T4 or T3 increases synthesis of NGF in mouse submandibular glands and brain [27–31]. Black and colleagues [27] demonstrated increased *NGF* mRNA production in neonatal mouse salivary glands for 24–72 h following a single intravenous injection of thyroid hormone (triiodothyronine, T3). These previous animal studies and our present investigation suggest a thyroid-hormone regulated transcription of the *NGF* gene that may account for some of the cases of detectable serum proNGF, although this observation requires validation in a larger cohort of hyperthyroid patients. We hypothesise that the detected proNGF is not of thyroidal origin, as it remained present in the serum of 91% of cases for which a paired post-thyroidectomy sample was available, but rather is likely to be secreted from an alternate site, such as salivary glands [32] under the regulation of thyroid hormone.

## Conclusions

In conclusion, these data show that proNGF is not a useful clinical biomarker of thyroid malignancies. From a translational perspective, it is important to report data on both biomarkers that show promises as well as those that are not clinically useful. In addition, the association between proNGF and hyperthyroidism that we have observed warrants further investigation to better understand the molecular and/or functional relationship between proNGF and hyperthyroidism.

## Supplementary information

**Additional file 1: Table S1.** (A-E): ProNGF ELISA Validation Experiments – Serum and Rinse. **A**: Effect of heterophilic antibody blockers (Ab) (BL-003-1000, Biosensis, Australia) on rate of positivity of serum proNGF levels. **B**: Spike and Recovery Experiments. **C**: Inter-plate Quality Control Samples **D**: Linearity of Dilution **E**: Linearity of Dilution of Biopsy Rinse Diluent.

**Additional file 2: Table S2.** Individual patient characteristics for cases with detectable serum proNGF.

**Additional file 3: Table S3.** Individual patient characteristics for cases with detectable biopsy proNGF. Age is presented as a range to preserve anonymity.

## Data Availability

All data generated or analysed during this study are included in this published article [and its supplementary information files].

## Abbreviations

ELISA: Enzyme-linked immunosorbent assay
FNA: Fine needle aspiration
NGF: Nerve growth factor
ProNGF: Precursor for nerve growth factor
QC: Quality control
TSH: Thyroid stimulating hormone
