# Supplementary material for: The precursor for nerve growth factor (proNGF) is not a serum or biopsy-rinse biomarker for thyroid cancer diagnosis
Source: BMC Endocr Disord. 2019 Nov 27;19:128. doi: 10.1186/s12902-019-0457-1 (PMC6882079; doi:10.1186/s12902-019-0457-1)

Additional Table 1 (A-E): ProNGF ELISA Validation Experiments – Serum and Rinse

1A: Effect of heterophilic antibody blockers (Ab) (BL-003-1000, Biosensis, Australia) on rate of positivity of serum proNGF levels. No changes were observed in performance of the standard curve with presence or absence of antibody blockers (data not shown).

Disease category	Serum samples assayed (n)	% of samples positive No Heterophilic Ab blockers	With Heterophilic Ab blockers	p-value
Thyroid cancer	20	45%	15%	0.04
Benign thyroid disease	20	30%	15%	0.25
No thyroid disease	18	44%	6%	0.006

1B: Spike and Recovery Experiments. 4 serum samples were assayed before and after a 2.5ng/mL spike (50ng/mL following adjustment for dilution) of recombinant proNGF (Biosensis, Australia) at 1:20 dilution, in the presence of Heterophilic Antibody Blockers (BL-003-1000).

Sample	ProNGF (ng/mL) Pre-spike	Post-spike	% recovery of spike
Sample A	2.7	66.7	128%
Sample B	0.0	47.8	96%
Sample C	0.1	41.0	82%
Sample D	0.0	40.1	80%
**Mean**			**96%**

1C: Inter-plate Quality Control Samples A quality-control solution (QC) was prepared by spiking 1:20 diluted proNGF-negative serum with 1.61ng/mL (32.15ng/mL after correction for dilution) recombinant proNGF with Heterophilic Antibody Blockers. Aliquots of the QC sample were included on each ELISA plate. Plate numbers reflect two different batches of ELISA plates.

Plate	1.1	1.2	1.3	1.4	1.5	1.6	2.1	2.2	2.3	2.4	2.5	Mean (SD)
proNGF (ng/mL)	39.9	31.9	34.5	41.4	41.1	29.3	20.1	29.0	29.5	18.6	32.2	**31.6 ± 7.3**
Percent Recovery	124%	99%	107%	129%	128%	91%	63%	90%	92%	58%	100%	**98 ± 22%**

For the biopsy rinse, a similar QC solution was prepared by spiking 1.6ng/mL recombinant proNGF into phosphate buffered saline with the addition of protease inhibitors and heterophilic antibody blockers (38µg/mL). Aliquots of the QC sample were included on each ELISA plate. Plate number represent different batches.

Plate	1.5	1.6	1.8	3.1	3.2	4.1	4.2	Mean (SD)
proNGF (ng/mL)	2.03	1.53	1.77	2.27	2.41	1.56	1.55	**1.87 ± 0.34**
Percent Recovery	127%	96%	111%	142%	151%	98%	97%	**117 ± 20%**

1D: Linearity of Dilution 3 serum samples positive for endogenous proNGF were assayed in serial dilution using Assay Diluent A (Biosensis, Australia) and heterophilic antibody blockers (BL-003-1000, Biosensis, Australia). The measured concentration at 1:20 dilution was defined as 100%. Reasonable linearity of dilution was observed. The zero values observed correspond to expected values below the limit of detection.

Sample	Endogenous ProNGF (ng/mL), corrected for dilution			
	1:10	1:20	1:40	1:80
Sample A	18.9 (109%)	17.3 (100%)	10.4 (60%)	14.6 (84%)
Sample B	2.6 (106%)	2.4 (100%)	0	0
Sample C	13.5 (124%)	10.9 (100%)	6.7 (62%)	0

1E: Linearity of Dilution of Biopsy Rinse Diluent

In addition to the spike and recovery experiments performed above, a 1 in 2 dilution series from 5ng/mL was prepared as the standard curve for biopsy rinse specimens using the same phosphate-buffered saline with the addition of protease inhibitors and heterophilic antibody blockers that was used to prepare the biopsy rinse specimens. 4 parameter logistic regression curves are shown below for the first three ELISA plates, showing excellent linearity of dilution.

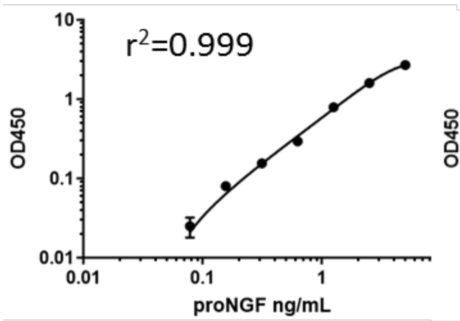

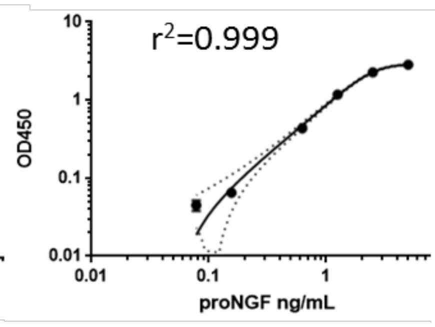

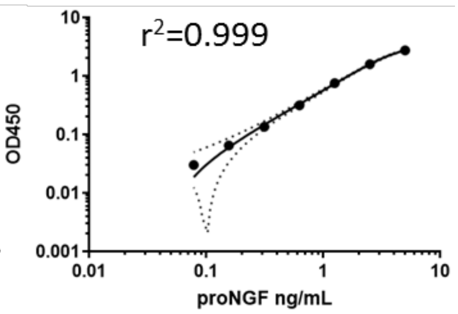

Supplement: Supplementary file 1 — Additional file 1: Table S1. (A-E): ProNGF ELISA Validation Experiments – Serum and Rinse. A: Effect of heterophilic antibody blockers (Ab) (BL-003-1000, Biosensis, Australia) on rate of positivity of serum proNGF levels. B: Spike and Recovery Experiments. C: Inter-plate Quality Control Samples D: Linearity of Dilution E: Linearity of Dilution of Biopsy Rinse Diluent. [file 12902_2019_457_MOESM1_ESM.pdf]
