# Supplementary material for: The precursor for nerve growth factor (proNGF) is not a serum or biopsy-rinse biomarker for thyroid cancer diagnosis
Source: BMC Endocr Disord. 2019 Nov 27;19:128. doi: 10.1186/s12902-019-0457-1 (PMC6882079; doi:10.1186/s12902-019-0457-1)
Supplement: Supplementary file 2 — Additional file 2: Table S2. Individual patient characteristics for cases with detectable serum proNGF. [file 12902_2019_457_MOESM2_ESM.pdf]

Additional Table 2: Individual patient characteristics for cases with detectable serum proNGF.

CASE	AGE	SEX	PRIMARY (SECONDARY) DIAGNOSIS	REASON FOR INVESTIGATION	SERUM PRONGF (ng/mL) INITIAL	POST-OT	TSH (mIU/L)	BASIS OF DIAGNOSIS
10087	51-60	F	Graves'	Treatment of GD	8.7	4.3	<0.01	Histopathology
10125	21-30	F	Graves'	Treatment of GD	4.3	2.8	<0.01	Histopathology
10140	31-40	M	Graves'	Treatment of GD	12.9	11.8	<0.01	Histopathology
10148	31-40	F	Graves'	Treatment of GD	1.6	2.3	<0.01	Histopathology
10166	21-30	F	Graves'	Treatment of GD	5.4	4.6	<0.01	Histopathology
10139	21-30	M	Graves'	Treatement of GD	1.0	.	0.01	Histopathology
10318	61-70	F	Toxic MNG	Hyperthyroid	12.6	.	0.14	Histopathology
10056	81-90	F	PTC	Recurrence	6	.	<0.01	Histopathology
10208	21-30	F	PTC (8mm, 5mm)	Treatment of GD	2.2	.	<0.01	Histopathology
10276	31-40	F	PTC (21mm)	Suspicious nodule	6.4	.	0.6	Histopathology
10101	81-90	F	PTC (15mm) (FA)	Suspicious nodule	9.3	1.8	0.77	Histopathology
10105	51-60	M	PTC (5mm) (MNG)	Compression	5.6	3.2	5.88	Histopathology
10124	41-50	M	HCC (35mm) (FA)	Suspicious nodule	7.6	2.3	1.37	Histopathology
10062	41-50	F	FA	Nodule	8	.	1.2	Histopathology
10463	61-70	F	FA	Nodule	14.7	.	1.6	Biopsy + USS
10173	71-80	F	MNG	Compression	1.3	.	0.47	Histopathology
10117	31-40	F	MNG	Compression	6.8	1.8	0.4	Histopathology
10410	51-60	F	MNG	Compression	2.9	.	1.3	Histopathology
10164	41-50	F	MNG	Compression	5.8	5.9	1.34	Histopathology
10340	41-50	M	MNG	Nodule	3.4	.	1.66	Biopsy + USS
10116	51-60	M	MNG	Compression	3.1	0.7	2.7	Histopathology
10465	41-50	F	MNG	Nodule	4.3	.	1.2	Biopsy + USS
10005	31-40	F	Benign nodule	Nodule	5.1	.	0.5	Biopsy + USS
10458	51-60	F	Benign nodule	Nodule	3.5	.	1.3	Biopsy + USS
10274	31-40	M	Benign nodule	Nodule	1.4	.	1.32	Biopsy + USS
10368	61-70	F	Benign nodule	Nodule	1.3	.	2.1	Biopsy + USS
10424	61-70	F	Benign nodule	Nodule	1.2	.	2.3	Biopsy + USS
10445	61-70	F	Normal	Completion HTx	8.7	.	0.66	Histopathology

FA: Follicular adenoma. MNG: Multinodular Goitre. GD: Graves' Disease. PTC: Papillary thyroid cancer. HCC: Hurthle cell cancer. USS: Neck ultrasound. HTx: Hemithyroidectomy. Initial: Mean of 6 replicates performed over a minimum of 2 separate assays. Post-OT: post thyroidectomy sample (2-14 days). Age is presented as a range to preserve patient anonymity.
