# Supplementary material for: The precursor for nerve growth factor (proNGF) is not a serum or biopsy-rinse biomarker for thyroid cancer diagnosis
Source: BMC Endocr Disord. 2019 Nov 27;19:128. doi: 10.1186/s12902-019-0457-1 (PMC6882079; doi:10.1186/s12902-019-0457-1)
Supplement: Supplementary file 3 — Additional file 3: Table S3. Individual patient characteristics for cases with detectable biopsy proNGF. Age is presented as a range to preserve anonymity. [file 12902_2019_457_MOESM3_ESM.pdf]

Additional Table 3: Individual patient characteristics for cases with detectable biopsy proNGF. Age is presented as a range to preserve anonymity.

CASE	AGE (years)	SEX	TSH (mIU/L)	BIOPSY PRONGF (ng/mL)	CYTOLOGY Bethesda Score	NODULE DIAGNOSIS	BASIS OF DIAGNOSIS
10534	71-80	F	0.87	0.1	6	Anaplastic thyroid cancer	Biopsy and USS
10018	81-90	F	2.8	0.2	2	Follicular thyroid cancer	Histopathology
10280	71-80	F	3	0.16	3	Follicular thyroid cancer	Histopathology
10343	51-60	M	2.37	0.58	4	Follicular thyroid cancer	Histopathology
10587	31-40	M	1.33	0.21	3	Follicular thyroid cancer	Histopathology
10060	81-90	F	0.86	1.79	2	Papillary thyroid cancer	Histopathology
10367	31-40	F	1.05	0.11	5	Papillary thyroid cancer	Histopathology
10564	41-50	F	5.2	0.19	4	Papillary thyroid cancer	Histopathology
10581	41-50	F	0.15	0.2	6	Papillary thyroid cancer	Histopathology
10589	51-60	F	0.7	0.33	5	Papillary thyroid cancer	Histopathology
10596	51-60	F	0.71	0.55	5	Papillary thyroid cancer	Histopathology
10544	61-70	M	1.4	0.25	6	Papillary thyroid cancer	Histopathology
10005	31-40	F	0.5	0.06	2	Benign nodule	Biopsy and USS
10368	51-60	F	2.1	0.05	2	Benign nodule	Biopsy and USS
10483	51-60	F	1.12	0.13	2	Benign nodule	Biopsy and USS
10486	41-50	F	1.06	0.15	2	Benign nodule	Biopsy and USS
10530	81-90	F	1	0.16	2	Benign nodule	Biopsy and USS
10531	61-70	F	1.9	0.15	2	Benign nodule	Biopsy and USS
10014	21-30	M	1.6	0.12	2	Benign nodule	Biopsy and USS
10502	81-90	M	1.83	0.1	2	Benign nodule	Biopsy and USS
10334	41-50	F	0.63	0.07	2	Benign nodule	Biopsy and USS
10402	41-50	F	2.2	0.15	2	Benign nodule	Biopsy and USS
10494	31-40	F	1.5	0.1	2	Benign nodule	Biopsy and USS
10504	21-30	F	.	0.08	2	Benign nodule	Biopsy and USS
10512	31-40	F	0.94	0.06	2	Benign nodule	Biopsy and USS
10524	71-80	F	0.72	0.1	2	Benign nodule	Biopsy and USS
10537	51-60	F	2.04	0.27	2	Benign nodule	Biopsy and USS

10547	31-40	F	2.9	0.16	2	Benign nodule	Biopsy and USS
10549	31-40	F	1	0.21	2	Benign nodule	Biopsy and USS
10550	61-70	F	0.6	0.09	2	Benign nodule	Biopsy and USS
10552	51-60	F	0.58	0.16	2	Benign nodule	Biopsy and USS
10553	71-80	F	1.5	0.32	2	Benign nodule	Biopsy and USS
10554	71-80	F	1.5	0.13	2	Benign nodule	Biopsy and USS
10555	51-60	F	2.86	0.12	2	Benign nodule	Biopsy and USS
10559	21-30	F	0.96	0.17	2	Benign nodule	Biopsy and USS
10560	41-50	F	2	0.11	2	Benign nodule	Biopsy and USS
10562	71-80	F	1.19	0.05	2	Benign nodule	Biopsy and USS
10565	51-60	F	2	0.24	2	Benign nodule	Biopsy and USS
10566	41-50	F	0.9	0.13	2	Benign nodule	Biopsy and USS
10569	71-80	F	2.6	0.21	2	Benign nodule	Biopsy and USS
10574	61-70	F	1.3	0.38	2	Benign nodule	Biopsy and USS
10575	61-70	F	2.1	0.16	2	Benign nodule	Biopsy and USS
10578	61-70	F	1.07	0.05	2	Benign nodule	Biopsy and USS
10558	31-40	F	0.94	0.13	2	Benign nodule	Biopsy and USS
10558	31-40	F	0.94	0.09	2	Benign nodule	Biopsy and USS
10355	51-60	M	0.57	0.06	2	Benign nodule	Biopsy and USS
10404	61-70	M	1.2	0.05	2	Benign nodule	Biopsy and USS
10462	61-70	M	1.1	0.13	2	Benign nodule	Biopsy and USS
10499	41-50	M	0.3	0.05	2	Benign nodule	Biopsy and USS
10538	31-40	M	1.35	0.25	2	Benign nodule	Biopsy and USS
10568	61-70	M	0.72	0.3	2	Benign nodule	Biopsy and USS
10572	61-70	M	0.92	0.05	2	Benign nodule	Biopsy and USS
10573	31-40	M	0.25	0.19	2	Benign nodule	Biopsy and USS
10587	31-40	M	1.33	0.17	2	Benign nodule	Biopsy and USS
10012	31-40	F	0.2	0.11	2	Benign nodule	Histopathology
10053	51-60	F	2.66	0.11	2	Benign nodule	Histopathology
10366	41-50	F	1.1	0.18	2	Benign nodule	Histopathology

10405	71-80	F	0.35	0.06	2	Benign nodule	Histopathology
10463	61-70	F	.	0.09	3	Benign nodule	Histopathology
10501	71-80	F	0.3	0.16	2	Benign nodule	Histopathology
10521	51-60	F	2.04	0.65	4	Benign nodule	Histopathology
10541	71-80	M	1.5	0.19	3	Benign nodule	Histopathology
10423	11-20	F	.	0.05	2	Follicular adenoma	Histopathology
10543	51-60	F	.	0.32	3	Follicular adenoma	Histopathology
10576	31-40	F	1.5	0.38	3	Follicular adenoma	Histopathology
10570	61-70	M	1.14	0.45	4	Hurthle cell adenoma	Biopsy and USS
10308	41-50	F	.	0.09	4	Hurthle cell adenoma	Histopathology
10377	31-40	F	1.41	0.05	2	Hurthle cell adenoma	Histopathology
10548	41-50	F	0.41	0.56	4	Hurthle cell adenoma	Histopathology
10597	51-60	F	2.1	0.15	1	Hurthle cell adenoma	Histopathology
10598	71-80	F	2.6	0.3	3	Hurthle cell adenoma	Histopathology
10481	51-60	F	3.4	0.21	2	Lymphocytic thyroiditis	Histopathology
10599	51-60	F	2.1	0.27	4	Lymphocytic thyroiditis	Histopathology
